# *Crotalus durissus terrificus* snake venom activates 3T3-L1 preadipocytes to release PGE_2_, which depends on COX-2 expression and is modulated by PGE_2_ EP2 receptor

**DOI:** 10.1007/s00204-025-04109-w

**Published:** 2025-06-23

**Authors:** Danilo Santos Teixeira, Rodrigo Maia-Marques, Catarina Teixeira

**Affiliations:** Laboratory of Pharmacology, Instituto Butantan, Sao Paulo, Brazil

**Keywords:** *Crotalus durissus* snake venom, Preadipocyte, Prostaglandin E_2_, PGE_2_ receptors

## Abstract

Snakebites are a worldwide public health problem with high-cost treatment in many countries. *Crotalus* genus snakes, commonly known as rattlesnakes, cause the most lethal snakebites in Brazil. *Crotalus* ssp envenomation is characterised by systemic neurotoxicity, myotoxicity, renal failure and minor local effects. Despite the knowledge of envenomation’s pathogenesis, the impact of *Crotalus* venom on adipose tissue (AT) cells is unknown. AT is an endocrine organ capable of releasing diverse immunomodulatory molecules, including prostaglandin E_2_ (PGE_2_). Herein, we investigated the effects of *Crotalus durissus terrificus* venom (CdtV) on preadipocytes in vitro, focussing on the release of PGE_2_ and mechanisms involved. CdtV (5 and 10 μg/mL) induced a marked release of PGE_2_ by preadipocytes (3–24 h) compared to controls. Pre-treatment of cells with SC-560 or NS-398, selective inhibitors for cyclooxygenase (COX)-1 and COX-2 enzymes, respectively, decreased CdtV-induced PGE_2_ release after 24 h. CdtV (1 and 10 μg/mL) did not change COX-1 protein expression by preadipocytes, but induced COX-2 protein expression at all time intervals evaluated. Pre-treating preadipocytes with AH6809, an inhibitor of the PGE_2_ EP2 receptor, significantly increased the CdtV-induced PGE_2_ release. EP1, EP3, or EP4 receptor antagonists did not change the CdtV-induced PGE_2_ release. Additionally, CdtV did not alter the EP2 receptor protein expression in preadipocytes. These findings demonstrate that CdtV activates preadipocytes to produce PGE_2_ via COX-1 activation and COX-2 protein expression and PGE_2_ triggers a negative feedback loop via EP2 for its own production. These results highlight AT as another target for CdtV that may contribute to envenomation.

## Introduction

Snakebites are a worldwide public health problem with high-cost treatment in many countries (Gutiérrez et al. 2011). The Viperidae and Elapidae families comprise clinically important groups of venomous snakes (Bucaretchi et al. 2016; da Silva et al. 2024). The Viperidae family is responsible for the majority of envenomation, whereas the genus *Crotalus* causes the highest number of deaths from snakebites in Brazil (Gutiérrez et al. 2009, 2010). The most important effects of envenomation by the *Crotalus* species are systemic neurotoxicity, myotoxicity, coagulopathy, and acute renal failure associated with myotoxicity, culminating in failure of end organs and death (Frare et al. 2019). Moreover, researchers have reported that *Crotalus* venom induces hypotension and bradycardia (Simões et al. 2021). In contrast to the *Bothrops* genus venoms (Viperidae family), which are proinflammatory, *Crotalus durissus terrificus* venom has unique features that does not induce an inflammatory response at the bite site (Baudou et al. 2023). Reports on clinical observations of CdtV envenomation victims indicate minor inflammatory signs and symptoms at the bite site, such as slight oedema and erythema as well as mild pain and local or regional paraesthesia that may persist for different periods of time (Rosenfeld 1971; De Carvalho et al. 2019). Systemic neuro- and myotoxicity induced by *C. durissus* ssp. bites have been associated with a high concentration of crotoxin, CdtV’s major component that can induce peripheral neuromuscular paralysis and cardiorespiratory failure, which are some of its main lethal effects (D Vaz de Melo et al. 2020). In addition to neurotoxicity, crotoxin induces the biosynthesis of the eicosanoids, such as lipoxin A_4_ (LXA_4_) via the lipoxygenase pathway as well as the prostaglandins E_2_, D_2_ and 15-d-PGJ_2_ by macrophages via the cyclooxygenase (COX)-1 pathway as well as cross talk with the endogenous Ca^2+^-independent PLA_2_ (Sampaio et al. 2006; Giannotti et al. 2017). The release of PGE_2_ by macrophages is also induced by the crude venom of CdtV (Sampaio et al. 2006).

PGE_2_ is an important mediator that modulates a series of physiological processes, including vascular tonus, blood pressure (Stock et al. 2001), kidney pressure (Wang et al. 2019), and gastric homeostasis (Takeuchi and Amagase 2018). PGE_2_ synthesis is regulated by key enzymes in the COX system. Moreover, PGE_2_’s wide-range effects are mediated by four receptor subtypes (EP1–4) (Sugimoto and Narumiya 2007), which differ in tissue distribution, ligand-binding affinity, and coupling to distinct intracellular signalling pathways. In pathophysiological conditions, however, systemic levels of PGE_2_ drastically increase. In this condition, PGE_2_ has been described as an important lipid mediator with dual effects during the inflammatory response. Notably, this mediator can positively or negatively modulate the inflammatory reaction depending on the microenvironment. On the inflammatory side, PGE_2_ potentiates the increase of vascular permeability induced by vasoactive amines and other mediators, thus leading to oedema formation. Regarding anti-inflammatory effects, researchers have demonstrated that PGE_2_ stimulates the release of the anti-inflammatory mediators interleukin-10 and LXA_4_ (MacKenzie et al. 2013; Loynes et al. 2018). In this sense CdtV inhibits the macrophages’ phagocytotic activity in vitro via production of LXA_4_ by these cells (Sampaio et al. 2006). Additionally, PGE_2_ has been associated with the nephrotoxic effect of *Crotalus durissus* ssp venom: *C. durissus cascavella* has been associated to kidney cell production of prostaglandins and other mediators. However, *Crotalus* venom’s ability to stimulate other PGE_2_ sources remains unknown. Regarding production of PGE_2_, the adipose tissue (AT) is largely known to produce and be regulated by this lipid mediator.

AT is widely distributed throughout the human body, producing and secreting an array of inflammatory mediators that contribute to the development and regulation of diverse inflammatory diseases. This is evidenced by AT’s participation in obesity, rheumatoid arthritis, and COVID-19 (Giles et al. 2018; Kawai et al. 2021; Yu et al. 2022). AT primarily comprises mature adipocytes and the stromal vascular fraction (SVF), which generates many of the mediators secreted by this tissue. Within the SVF, preadipocytes (the precursors of mature adipocytes), account for up to 50% of human AT cells (Dordevic et al. 2014). These cells are more active than mature adipocytes due to the high metabolic activity which is needed to support proliferation and differentiation into mature adipocytes as well as adaptation to the microenvironment. Moreover, preadipocytes have important immune and haemostatic functions (Charrière et al. 2006; Dashty et al. 2012). Despite the AT’s importance to physiological homeostasis and pathophysiological conditions, no evidence indicates that AT cells are activated by *Crotalus* venom; therefore, we hypothesise that CdtV activates AT to produce pro and/or anti-inflammatory mediators. Furthermore, adipocytes can produce and release mediators which modulate the inflammatory response. In this study we investigated the effects of CdtV on preadipocytes in culture, focussing on the release of PGE_2_ and the mechanisms involved in this effect. The findings reported herein demonstrate that CdtV activates preadipocytes to produce PGE_2_ by a mechanism dependent on COX-1 activation and COX-2 protein expression. Additionally, the PGE_2_ released by this venom stimulates a negative feedback loop via EP2 for its own production.

## Material and methods

### Venom, chemicals and reagents

*Crotalus durissus terrificus* venom (CdtV) was collected, lyophilized, identified and provided by the Herpetology Laboratory of Instituto Butantan. The venom batches used were tested for endotoxin contamination using the quantitative limulus amoebocyte lysate test (Takayama et al. 1994), which revealed undetectable levels of endotoxin (< 0.125 EU/mL). The venom was reconstituted in sterile PBS and filtered immediately before use. L-glutamine was purchased from USB (Cleveland, OH, USA). Dulbecco’s Modified Eagle Medium (DMEM) and Foetal Bovine Serum (FBS) were purchased from Life Technologies (São Paulo, SP, Brazil); gentamicin was purchased from Schering-Plough (Whitehouse Station, NJ, USA); 3-(4,5-dimethylthiazol-2-yl)-2,5-diphenyltetrazolium bromide (MTT), dimethyl sulfoxide (DMSO) and mouse anti-β-actin monoclonal antibody were purchased from Sigma-Aldrich (St. Louis, MO, USA); and polyclonal antibody against COX-1, the PGE_2_ enzyme immunoassay kit and compounds SC-560, NS-398, SC-19220, AH6890, L-798106 and GW 627368X were purchased from Cayman Chemical Company (Ann Arbor, MI, USA). Polyclonal antibody against COX-2, HRP-conjugated anti-mouse secondary antibody were purchased from Thermo Fisher (Waltham, Massachusetts, USA). HRP-conjugated anti-rabbit secondary antibody and nitrocellulose membrane were purchased from GE Healthcare (Buckinghamshire, UK).

### 3T3-L1 cell culture

3T3-L1 murine preadipocytes obtained from the American Type Culture Collection were cultured in Dulbecco’s Modified Eagle Medium (DMEM) supplemented with 10% (v/v) FBS until confluence. Before stimulation with CdtV, FBS in the media was replaced by 0.2% Bovine Serum Albumin (BSA).

### Quantification of PGE_2_

Quantification of PGE_2_ was performed in the supernatants collected from cell cultures by enzyme immunoassay (EIA) using a commercially available kit (Cayman Chemicals, ThermoFisher). The tests were performed according to the supplier’s specifications. Concentrations were estimated from the standard curve and represented in pg/mL.

### Pharmacological interventions

To evaluate the participation of COX-1, COX-2 and each PGE_2_ receptor subtype in the CdtV-induced effects, pharmacological interventions were performed with selective inhibitors or antagonists in concentrations described in the literature (Choi et al. 2008; Lin et al. 2008, 2009; Chen et al. 2012): 1 μM SC-560 (COX-1 inhibitor, 1 h before CdtV); 1 μM NS-398 (COX-2 inhibitor, 1 h before CdtV); 10 μM SC-19220 (EP1 receptor antagonist, 1 h before CdtV); 10 μM AH 6809 (EP2 receptor antagonist, 1 h before CdtV); 1 μM L-798,106 (EP3 receptor antagonist, 1 h before CdtV); 10 μM GW 627368X (EP4 receptor antagonist, 1 h before CdtV). Some of the used compounds were prepared in DMSO at concentration lower than 1%. Cells treated with the inhibitors were analysed for viability by the MTT colorimetric assay. No significant changes in cell viability were registered with any of the above agents or vehicles at the concentrations used.

### Western blotting

The protein content of COX-1, COX-2 and EP2 receptor was determined in cell lysates by western blotting. For this purpose, the cells incubated or not with CdtV were lysed by adding 100 μL/well of Laemmli buffer (0.5 M Tris–HCl, 20% SDS, 1% glycerol, 1 M β-mercaptoethanol, 0.1% bromophenol blue) and boiled at 100 °C for 10 min. Samples were resolved by SDS-PAGE (12% bis-acrylamide gel) electrophoresis. The proteins were transferred to a nitrocellulose membrane with a Mini Trans-Blot (Bio-Rad Laboratories, Richmond, CA, USA). The membranes were blocked for 1 h with 5% non-fat dry milk in Tris-buffered saline Tween 20 (20 mM Tris, 100 mM NaCl and 0.5% Tween 20, pH 7.2) and incubated overnight at 4 °C with COX-1, COX-2 or EP2 primary antibodies (1:1000 dilution) and for 1 h at room temperature with the β-actin primary antibody (1:3000 dilution). Next, the membranes were washed and incubated with the appropriate secondary antibody conjugated to horseradish peroxidase. Immunoreactive bands were detected using an entry-level peroxidase substrate for enhanced chemiluminescence (Pierce ECL Western Blotting Substrate) according to the manufacturer’s instructions (Thermo Fisher Scientific, Waltham, MA, USA). Band images were captured with an ImageQuant LAS 4000 mini biomolecular imager (GE Healthcare) and analyzed with ImageQuant TL software (GE Healthcare).

### Statistical analysis

The results were expressed as mean + standard error of the mean (S.E.M.). One-way or Two-way analysis of variance (ANOVA) were used, followed by multiple comparisons with the Bonferroni post-test. The normality and homoscedasticity of all samples were checked previously. The data were analysed with GraphPad Prism 8.0.1 (GraphPad, San Diego, CA, USA). A significance level of p < 0.05 was adopted.

## Results

### CdtV stimulates the release of PGE_2_ by preadipocytes

**Fig. 1:**
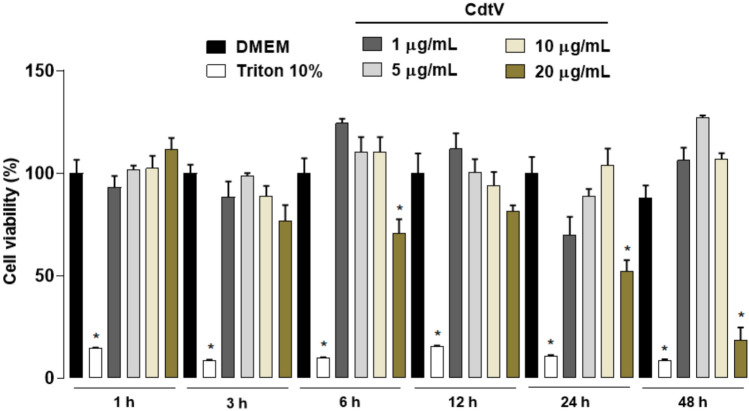
Time course of CdtV effect on viability of preadipocyte cell culture. 3T3-L1 preadipocytes were incubated with CdtV (1–20 μg/mL), DMEM (negative control), or 10% Triton (positive control) for 1 up to 48 h. Metabolic activity was assessed by the MTT assay. Results are expressed as mean + S.E.M. of 3 independent assays (n = 4). * *p* < 0.05 *vs* DMEM group of the respective experimental time (ANOVA, Bonferroni’s post-test)

**Fig. 2:**
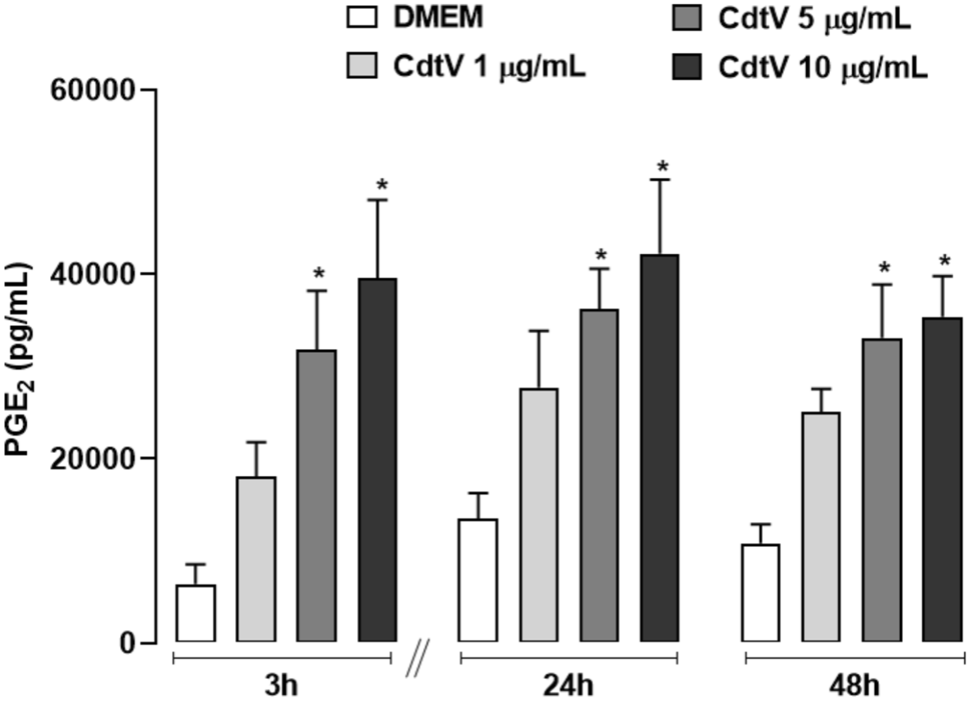
Release of PGE_2_ by preadipocytes stimulated with CdtV. 3T3-L1 preadipocytes were incubated with CdtV (1, 5 or 10 μg/mL) or DMEM for 3, 24 or 48 h. Concentration of PGE_2_ in cell culture supernatants was evaluated by EIA. Results represent the time course of PGE_2_ release induced by CdtV and are expressed as mean + S.E.M. of 3 independent assays (n = 4). * *p* < 0.05 *vs* DMEM (ANOVA, Bonferroni’s post-test)

PGE_2_ is a mediator relevant to both physiological and pathological processes. In AT, it is released by constitutive cells to mediate preadipocyte differentiation. However, to date, researchers do not know whether CdtV can activate this tissue to synthesise PGE_2_. Our preliminary results indicate that CdtV doses up to 10 μg/mL do not affect preadipocyte cell culture viability (Fig. 1). Hence, 10 μg/mL is defined as the maximum non-cytotoxic concentration and is used in later steps. To investigate whether CdtV promotes PGE_2_ release by preadipocytes, this venom was added (1, 5, or 10 μg/mL) to the cell culture. At selected time intervals, PGE_2_ concentrations were evaluated in the cell culture supernatants. Figure 2 demonstrates that CdtV at concentrations of 5 and 10 μg/mL induced a marked release of PGE_2_ by preadipocytes at 3, 24, and 48 h after incubation compared to the respective negative control group. This result demonstrates that CdtV can activate AT to release PGE_2_.

### The COX enzymatic system participates in the CdtV-induced PGE_2_ release by preadipocytes

**Fig. 3:**
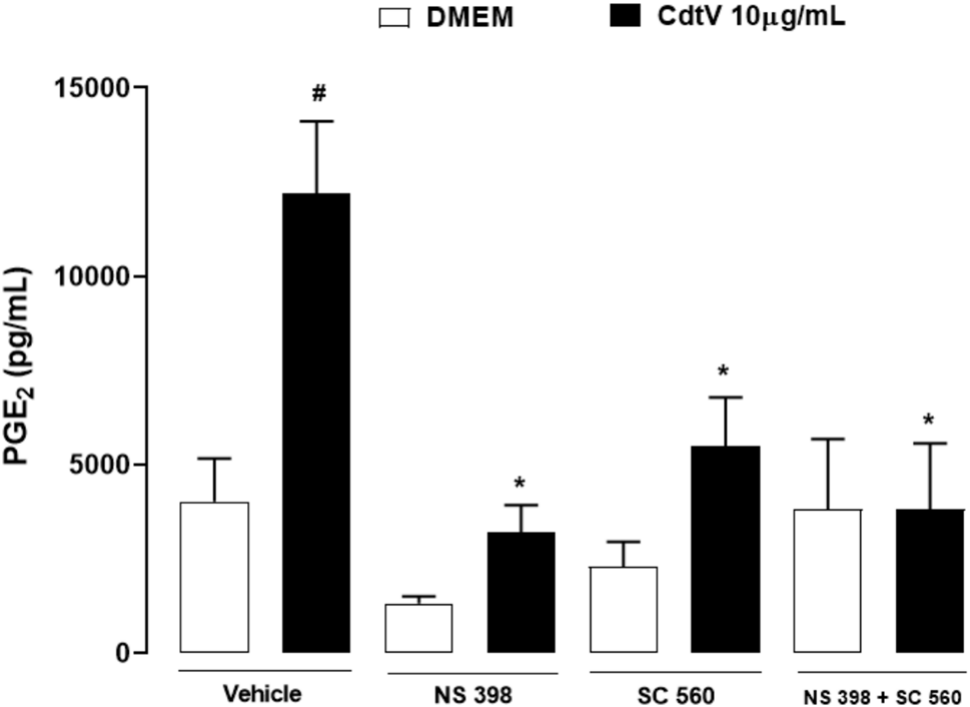
Participation of COX-1 and -2 in PGE_2_ release induced by CdtV in preadipocytes. 3T3-L1 preadipocytes were pretreated with the COX-1 and COX-2 inhibitors SC-560 and NS-398, respectively, or vehicle (DMSO < 1%) for 1 h. After this, the cells were stimulated with CdtV (10 μg/mL) or DMEM for 24 h. Levels of PGE_2_ present in cell culture supernatants were quantified by EIA. Results are expressed as mean + S.E.M. (n = 4). ^#^ *p* < 0.05 *vs* negative control (vehicle + DMEM); * *p* < 0.05 *vs* positive control (vehicle + CdtV) (ANOVA, Bonferroni’s post-test)

**Fig. 4:**
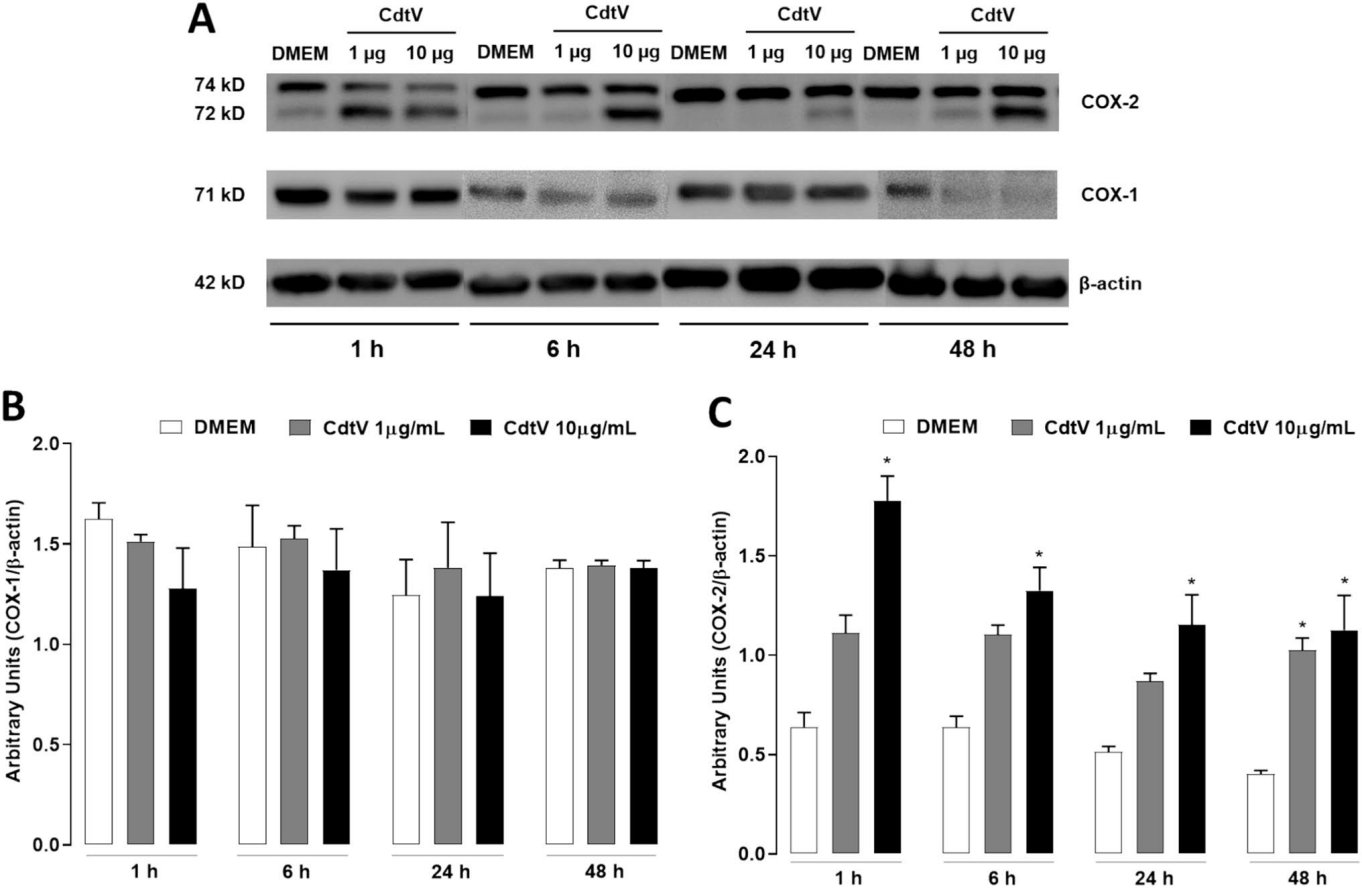
Effect of CdtV on COX-1 and COX-2 protein expression in preadipocytes. 3T3-L1 preadipocytes were stimulated with CdtV (1 or 10 μg/mL) or DMEM for 1, 6, 24 or 48 h. COXs protein expression was assessed by western blotting. **A** Immunoreactive bands of COX-2, COX-1 and β-actin (loading control). Representative blots were cropped from the full-length blots. **B** Densitometric analysis of immunoreactive bands of COX-1 and **C** COX-2. Results are expressed as mean + S.E.M (n = 4). * *p* < 0.05 *vs* control (ANOVA, Bonferroni’s post-test)

Prostanoid production is determined by a cascade of key enzymes; the first essential step is the modification of arachidonic acid by the COX system, which comprises at least four isotypes of COXs; COX-1(constitutive isoform) and COX-2 (constitutive and inducible isoform) are the most studied. We investigated whether COX-1 and -2 enzymes are involved in the PGE_2_ release, which is induced by CdtV in preadipocytes. For this, preadipocytes were preincubated (1 h) with SC-560 or NS-398, which are selective inhibitors of the isoforms COX-1 and COX-2, respectively, or with inhibitors vehicle (DMSO < 1%), followed by a 24-h incubation with CdtV. Then, PGE_2_ was quantified in cell culture supernatants. Figure 3 demonstrates that CdtV induced a marked release of PGE_2_ from preadipocytes in the vehicle group compared to the negative control group (DMEM plus vehicle). Cells that were pre-treated with pharmacological COX enzyme inhibitors and incubated with DMEM did not release PGE_2_. However, cells that were pre-treated with NS-398, SC-560, or both, demonstrated a decrease of PGE_2_ release after 24 h of incubation with CdtV compared to the CdtV plus vehicle group. These data indicate that both COX-1 and -2 contribute to PGE_2_ production induced by CdtV. On these bases, we examined the effects of CdtV on the protein expression of these enzymes. Immunoreactive bands showed in Fig. 4A demonstrate that CdtV did not change COX-1 protein expression by preadipocytes. The densitometric analysis of these bands, seen in Fig. 4B, confirms Fig. 4A, demonstrating that CdtV did not alter COX-1 protein expression in any concentration (1 or 10 μg/mL) or time interval studied, as expected. Conversely, Figs. 4A and C demonstrate that CdtV at the lower concentration (1 μg/mL) stimulated COX-2 protein expression only after 48 h of incubation, although the higher concentration (10 μg/mL) caused this effect at all time intervals observed. Together, these data indicate that COX-1 and COX-2 mediate PGE_2_ production, which is induced by CdtV and that this venom promotes an early COX-2 protein expression, which can contribute to the mechanism involved in the increased release of PGE_2_ provoked by this venom.

### EP2 receptor regulates the PGE_2_ release induced by CdtV

**Fig. 5:**
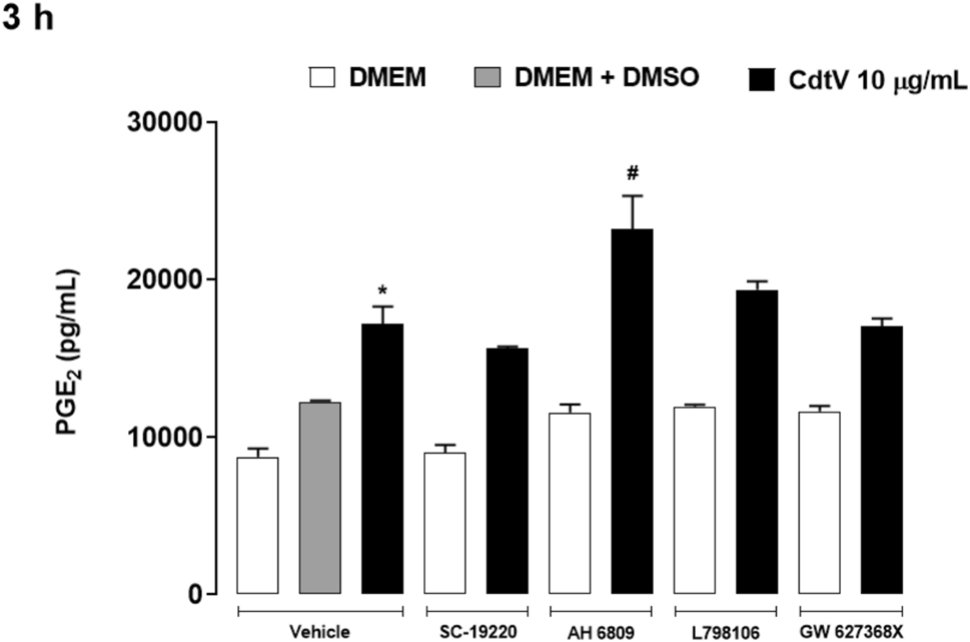
Participation of EP receptors in the release of PGE_2_ induced by CdtV in preadipocytes. 3T3-L1 preadipocytes were treated with 10 μM SC-19220 (EP1 receptor antagonist), 10 μM AH6809 (EP2 receptor antagonist), 1 μM L-798106 (EP3 receptor antagonist), 10 μM AH23848 (EP4 receptor antagonist) or vehicle (DMSO < 1%) for 1 h, and then stimulated with CdtV (10 μg/mL) for 3 h. Concentrations of PGE_2_ in cell culture supernatants was quantified by EIA. Results are expressed as mean + S.E.M. (n = 4). * *p* < 0.05 *vs* negative control (vehicle + DMEM); ^#^ *p* < 0.05 *vs* positive control (vehicle + CdtV) (ANOVA, Bonferroni’s post-test)

**Fig. 6:**
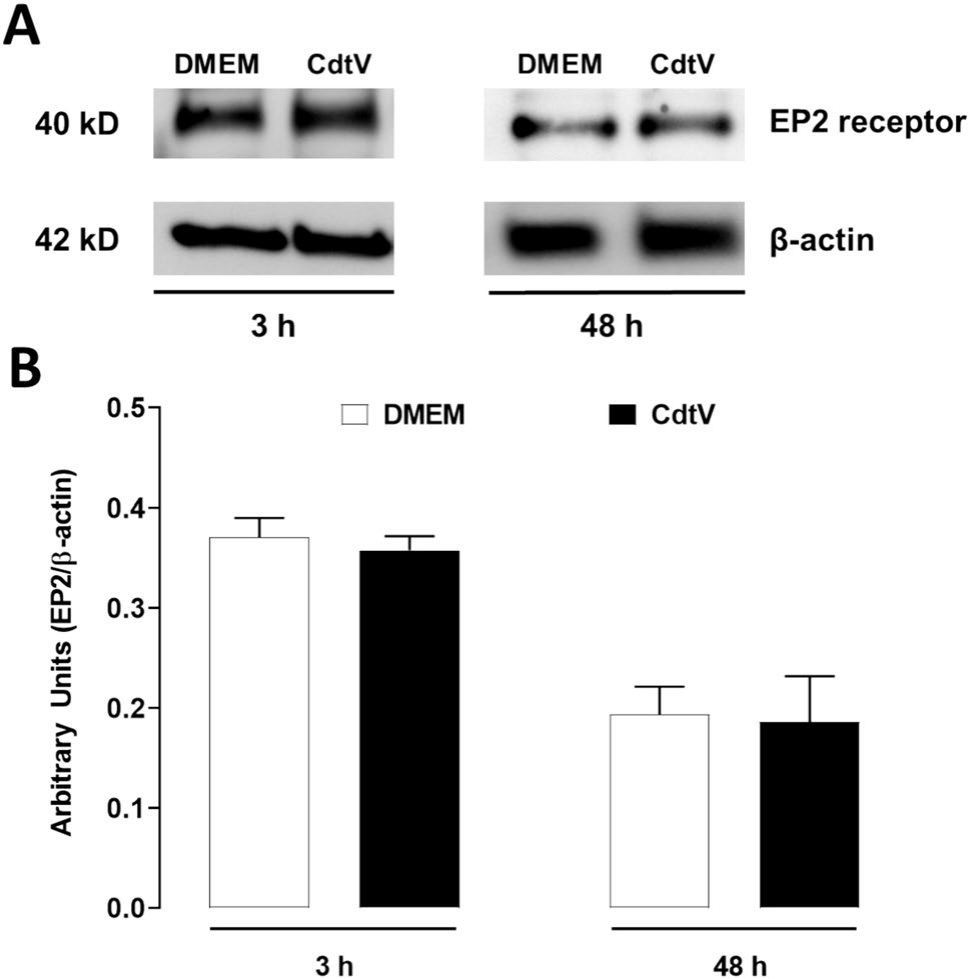
Effect of CdtV on EP2 protein expression in preadipocytes. 3T3-L1 preadipocytes were stimulated with CdtV (10 μg/mL) or DMEM for 3 or 48 h. EP2 protein expression was assessed by western blotting. **A** Immunoreactive bands of EP2 and β-actin (loading control). Representative blots were cropped from the full-length blots. **B** Densitometric analysis of immunoreactive bands of EP2. Results are expressed as mean + S.E.M (n = 4)

The effects initiated by PGE_2_ are due to its binding to a specific receptor with four subtypes: EP1–4. In addition to transducing PGE_2_ effects, these receptors modulate PGE_2_ release depending on the cell microenvironment and stimulus. Since CdtV induces PGE_2_ release by preadipocytes, we investigated EP receptors participation in this event. Figure 5 demonstrates that preadipocytes pretreated with the vehicle and incubated with CdtV displayed a marked PGE_2_ release. Furthermore, the EP receptor antagonists per se did not induce PGE_2_ release by these cells. The cells that were pretreated with AH6809, an inhibitor of the EP2 receptor, and then incubated with CdtV released more PGE_2_ compared to the vehicle plus venom group. However, pretreatment with EP1, EP3, or EP4 antagonists did not change the PGE_2_ release induced by CdtV. We further investigated whether CdtV could promote changes in EP2 protein expression. Figure 6 indicates that the venom did not alter the EP2 protein expression in preadipocytes. Together, these results indicate that the EP2 receptor modulates the release of PGE_2_ induced by CdtV in preadipocytes, thereby triggering a negative feedback loop for production of this mediator. This effect is not dependent on the increase of EP2 protein expression.

## Discussion

CdtV induces systemic neurotoxic, myotoxic, nephrotoxic and cardiovascular effects but lacks relevant local effects. The clinical importance and gravity of *Crotalus* envenomation in Latin America and the considerable number of studies on envenomation pathophysiology have not translated to a complete characterisation of the tissues targeted by crotalic venoms. Herein, we demonstrate for the first time that CdtV stimulates preadipocytes, leading to the release of PGE_2_, an important mediator of AT physiology that also fulfils dual roles during inflammatory responses. This result evidences a new source of this mediator upon CdtV stimulus and points to AT as another target for crotalic venom. Despite the critical differences between venoms from *Crotalus* and *Bothrops* genera in their components and biological actions, our results align with previous data that demonstrates the ability of *Bothrops* snake venom to activate AT (Maia-Marques et al. 2022); this reinforces the importance of AT in the response to snake envenomation and suggests AT as a common target for venoms of the Viperidae family.

AT is an endocrine organ that influences distant tissues and other organs; consequently, it is plausible to suggest that CdtV activation of this tissue impacts the systemic effects of this venom. In this context, the PGE_2_ release demonstrated here aligns with previous data showing that PGE_2_ contributes to CdtV’s systemic effects. Indeed, PGE_2_’s participation in the anti-inflammatory effect of crotoxin was demonstrated in an experimental model of acute intestinal inflammation (De Almeida et al. 2015). However, PGE_2_ is also associated with increased vascular permeability induced by the same toxin in lung parenchyma (Sartim et al. 2020). PGE_2_ is an important lipid mediator implicated in the development and regulation of inflammation and adipocyte differentiation. An essential step in PGE_2_ synthesis involves metabolising free arachidonic acid from cell membranes by COX enzymes, which generate intermediate products that are then processed by tissue-specific terminal synthases, leading to PGE_2_ formation. COX-1 and COX-2 are the most studied isoforms of this enzyme group; while COX-1 is constitutively expressed in many tissues, COX-2 is constitutively expressed in some tissues but is inducible in inflammatory processes. Both isoforms are responsible for producing prostaglandins in several physiological and pathological conditions.

To understand the mechanisms involved in CdtV-induced PGE_2_ production, we used pharmacological approaches. Our results demonstrating that the pharmacological inhibition of COX-1 and COX-2 isoforms diminished the preadipocyte release of PGE_2_ that was stimulated by CdtV, indicate that both isoforms contribute to this effect. In this context, the finding that CdtV induces COX-2 expression in preadipocytes adds important information regarding the molecular mechanism triggered by CdtV that leads to PGE_2_ production in preadipocytes. This is the first time that researchers have demonstrated that the crude venom of *C. d. terrificus* induces COX-2 protein expression. Our results agree with reports that CB, a major phospholipase A_2_ of CdtV, increases COX-2 gene expression in the lung tissue of mice (Sartim et al. 2020) and COX-2 protein expression in myoblasts in culture (Silva et al. 2021). Conversely, Moreira et al. (2007) demonstrated that CdtV did not induce COX-2 expression, but increased COX-1 activity in macrophages in culture, thus suggesting that CdtV's activation of PGE_2_’s synthetic pathways is associated with the cell type and/or function. Hence, our results highlight the preadipocytes as another cell target for this venom.

The physiological and pathophysiological effects initiated by PGE_2_ occur through its binding to four subtypes of G protein-coupled receptors, known as EP1–4. These receptors also regulate PGE_2_ release depending on the tissue and cell microenvironment (Golden et al. 2020). The results, which portray that pharmacologically inhibiting EP2 receptors increases CdtV-induced PGE_2_ production, point out the participation of this receptor subtype as a negative modulator of CdtV-induced PGE_2_ release. This regulation was not observed when using EP1, EP3, or EP4 receptor inhibitors. Thus, under CdtV’s effect, the autocrine action of PGE_2_ released by preadipocytes activates a negative feedback loop on its own production. It is worth noting that this negative modulation occurred during the initial venom action period (3 h) when a substantial release of this mediator was seen, which was not sustained after 48 h. However, we previously demonstrated that *Bothrops* venom can induce positive regulation of PGE_2_ production via EP receptors. Maia-Marques et al. (2022) showed that PGE_2_ released by preadipocytes that were stimulated with *B. moojeni* snake venom amplifies its own release by launching a positive feedback loop via the EP1 receptor. Additionally, stimulating preadipocytes with a PLA_2_ isolated from *B. asper* snake venom led to a similar PGE_2_ positive feedback mechanism by engaging the EP4 receptor (Leiguez et al. 2020). A PGE_2_ positive feedback loop was also caused by a snake venom metalloproteinase, BaP1, via the EP4 receptor in murine synoviocytes (Viana et al. 2020). Notably, EP receptor engagement by *Bothrops* venom or its toxins begins positive feedback for PGE_2_ release, whereas EP receptor activation by *Crotalus* venom results in negative feedback for the release of this mediator. In sum, our data suggest that EP receptors’ modulation of PGE_2_ release is related to the venom composition and origin (genus and species). However, we cannot eliminate the importance of the cell type for different PGE_2_ synthesis modulation that is initiated by EP receptors on different venoms.

Together, the data presented point out that CdtV stimulates preadipocytes to produce lipid mediators, such as PGE_2_. This effect depends on COX-1 activation and COX-2 expression. Additionally, in preadipocytes stimulated by CdtV, the activation of the EP2 receptor by autocrine PGE_2_ action begins a negative feedback loop. This is the first demonstration that CdtV activates AT cells. Therefore, our findings led to the discovery of another target cell and, consequently, another target tissue for CdtV. Since AT modulates many physiological and pathophysiological processes in the organism, our findings reveal that this tissue can be a source of lipid mediators during *Crotalus* envenomation.

## Data Availability

Access to the data is available on request.

## References

[CR1] Baudou FG, Gutiérrez JM, Rodríguez JP (2023) Immune response to neurotoxic South American snake venoms. Toxicon. 10.1016/j.toxicon.2023.107300 37757959 10.1016/j.toxicon.2023.107300

[CR2] Bucaretchi F, De Capitani EM, Vieira RJ et al (2016) Coral snake bites (Micrurus spp.) in Brazil: a review of literature reports. Clin Toxicol 54:222–234. 10.3109/15563650.2015.1135337 10.3109/15563650.2015.1135337 26808120

[CR3] Charrière GM, Cousin B, Arnaud E et al (2006) Macrophage characteristics of stem cells revealed by transcriptome profiling. Exp Cell Res 312:3205–3214. 10.1016/j.yexcr.2006.06.034 16934250 10.1016/j.yexcr.2006.06.034

[CR4] Chen L, Miao Y, Zhang Y et al (2012) Inactivation of the E-prostanoid 3 receptor attenuates the angiotensin II pressor response via decreasing arterial contractility. Arterioscler Thromb Vasc Biol 32:3024–3032. 10.1161/ATVBAHA.112.254052 23065824 10.1161/ATVBAHA.112.254052 PMC3565847

[CR5] Choi HC, Kim HS, Lee KY et al (2008) NS-398, a selective COX-2 inhibitor, inhibits proliferation of IL-1β-stimulated vascular smooth muscle cells by induction of ΗΟ-1. Biochem Biophys Res Commun 376:753–757. 10.1016/j.bbrc.2008.09.056 18809379 10.1016/j.bbrc.2008.09.056

[CR6] D Vaz de Melo P, de Almeida Lima S, Araújo P et al (2020) Immunoprotection against lethal effects of Crotalus durissus snake venom elicited by synthetic epitopes trapped in liposomes. Int J Biol Macromol 161:299–307. 10.1016/j.ijbiomac.2020.05.171 32464201 10.1016/j.ijbiomac.2020.05.171

[CR7] da Silva FFB, de Andrade MT, Siqueira-Silva T et al (2024) Predicting the drivers of Bothrops snakebite incidence across Brazil: a spatial analysis. Toxicon. 10.1016/j.toxicon.2024.108107 39343148 10.1016/j.toxicon.2024.108107

[CR8] Dashty M, Akbarkhanzadeh V, Zeebregts CJ et al (2012) Characterization of coagulation factor synthesis in nine human primary cell types. Sci Rep 2:1–9. 10.1038/srep00787 10.1038/srep00787 PMC3494008 23145311

[CR9] De Almeida CS, Andrade-Oliveira V, Câmara NOS et al (2015) Crotoxin from Crotalus durissus terrificus is able to down-modulate the acute intestinal inflammation in mice. PLoS ONE 10:1–19. 10.1371/journal.pone.0121427 10.1371/journal.pone.0121427 PMC4390225 25853847

[CR10] De Carvalho LH, Teixeira LF, Zaqueo KD et al (2019) Local and systemic effects caused by Crotalus durissus terrificus, Crotalus durissus collilineatus, and Crotalus durissus cascavella snake venoms in swiss mice. Rev Soc Bras Med Trop. 10.1590/0037-8682-0526-2018 31508780 10.1590/0037-8682-0526-2018

[CR11] Dordevic AL, Konstantopoulos N, Cameron-Smith D (2014) 3T3-L1 preadipocytes exhibit heightened monocyte-chemoattractant protein-1 response to acute fatty acid exposure. PLoS ONE 9:1–8. 10.1371/journal.pone.0099382 10.1371/journal.pone.0099382 PMC4049800 24911931

[CR12] Frare BT, Resende YKS, Dornelas BDC et al (2019) Clinical, laboratory, and therapeutic aspects of Crotalus durissus (South American Rattlesnake) victims: a literature review. Biomed Res Int. 10.1155/2019/1345923 31467868 10.1155/2019/1345923 PMC6699371

[CR13] Giannotti KC, Leiguez E, De CAEZ et al (2017) A snake venom group IIA PLA2 with immunomodulatory activity induces formation of lipid droplets containing 15-d-PGJ2 in macrophages. Sci Rep 7:1–15. 10.1038/s41598-017-04498-8 28642580 10.1038/s41598-017-04498-8 PMC5481388

[CR14] Giles JT, Ferrante AW, Broderick R et al (2018) Adipose tissue macrophages in rheumatoid arthritis: prevalence, disease-related indicators, and associations with cardiometabolic risk factors. Arthritis Care Res 70:175–184. 10.1002/acr.23253 10.1002/acr.23253 28388816

[CR15] Golden J, Illingworth L, Kavarian P et al (2020) EP2 receptor blockade attenuates COX-2 upregulation during intestinal inflammation. Shock 54:394–401. 10.1097/SHK.0000000000001444 31490357 10.1097/SHK.0000000000001444 PMC7051888

[CR16] Gutiérrez JM, Lomonte B, León G et al (2009) Snake venomics and antivenomics: proteomic tools in the design and control of antivenoms for the treatment of snakebite envenoming. J Proteomics 72:165–182. 10.1016/j.jprot.2009.01.008 19344652 10.1016/j.jprot.2009.01.008

[CR17] Gutiérrez JM, Williams D, Fan HW, Warrell DA (2010) Snakebite envenoming from a global perspective: towards an integrated approach. Toxicon 56:1223–1235. 10.1016/j.toxicon.2009.11.020 19951718 10.1016/j.toxicon.2009.11.020

[CR18] Gutiérrez JM, León G, Lomonte B, Angulo Y (2011) Antivenoms for snakebite envenomings. Inflamm Allergy Drug Targets 10:369–380. 10.2174/187152811797200669 21745181 10.2174/187152811797200669

[CR19] Kawai T, Autieri MV, Scalia R (2021) Adipose tissue inflammation and metabolic dysfunction in obesity. Am J Physiol Cell Physiol 320:C375–C391. 10.1152/ajpcell.00379.2020 33356944 10.1152/ajpcell.00379.2020 PMC8294624

[CR20] Leiguez E, Motta P, Maia Marques R et al (2020) A representative GIIA phospholipase A2 activates preadipocytes to produce inflammatory mediators implicated in obesity development. Biomolecules 10:1593. 10.3390/biom10121593 33255269 10.3390/biom10121593 PMC7760919

[CR21] Lin YS, Hsieh M, Lee YJ et al (2008) AH23848 accelerates inducible nitric oxide synthase degradation through attenuation of cAMP signaling in glomerular mesangial cells. Nitric Oxide Biol Chem 18:93–104. 10.1016/j.niox.2007.10.005 10.1016/j.niox.2007.10.005 18039475

[CR22] Lin CC, Lin WN, Wang WJ et al (2009) Functional coupling expression of COX-2 and cPLA2 induced by ATP in rat vascular smooth muscle cells: role of ERK1/2, p38 MAPK, and NF-κB. Cardiovasc Res 82:522–531. 10.1093/cvr/cvp069 19233864 10.1093/cvr/cvp069

[CR23] Loynes CA, Lee JA, Robertson AL et al (2018) PGE2 production at sites of tissue injury promotes an anti-inflammatory neutrophil phenotype and determines the outcome of inflammation resolution in vivo. Sci Adv. 10.1126/sciadv.aar8320 30191175 10.1126/sciadv.aar8320 PMC6124908

[CR24] MacKenzie KF, Clark K, Naqvi S et al (2013) PGE 2 induces macrophage IL-10 production and a regulatory-like phenotype via a protein kinase A-SIK–CRTC3 pathway. J Immunol 190:565–577. 10.4049/jimmunol.1202462 23241891 10.4049/jimmunol.1202462 PMC3620524

[CR25] Maia-Marques R, Teixeira DS, Janovits PM et al (2022) Bothrops moojeni snake venom induces an inflammatory response in preadipocytes: insights into a new aspect of envenomation. PLoS Negl Trop Dis 16:e0010658. 10.1371/journal.pntd.0010658 35939519 10.1371/journal.pntd.0010658 PMC9359566

[CR26] Moreira V, Zamuner SR, Wallace JL, Teixeira C de FP (2007) Bothrops jararaca and Crotalus durissus terrificus venoms elicit distinct responses regarding to production of prostaglandins E2 and D2, and expression of cyclooxygenases. Toxicon 49:615–624. 10.1016/j.toxicon.2006.09.006 10.1016/j.toxicon.2006.09.006 17241651

[CR27] Rosenfeld G (1971) Symptomatology, pathology, and treatment of snake bites in South America. In: Venomous animals and their venoms. Elsevier. pp 345–384

[CR28] Sampaio SC, Alba-Loureiro TC, Brigatte P et al (2006) Lipoxygenase-derived eicosanoids are involved in the inhibitory effect of Crotalus durissus terrificus venom or crotoxin on rat macrophage phagocytosis. Toxicon 47:313–321. 10.1016/j.toxicon.2005.11.008 16373074 10.1016/j.toxicon.2005.11.008

[CR29] Sartim MA, Souza COS, Diniz CRAF et al (2020) Crotoxin-induced mice lung impairment: role of nicotinic acetylcholine receptors and cox-derived prostanoids. Biomolecules. 10.3390/biom10050794 32443924 10.3390/biom10050794 PMC7277605

[CR30] Silva NC, Alvarez AM, DeOcesano-Pereira C et al (2021) Catalytically active phospholipase A2 myotoxin from Crotalus durissus terrificus induces proliferation and differentiation of myoblasts dependent on prostaglandins produced by both COX-1 and COX-2 pathways. Int J Biol Macromol 187:603–613. 10.1016/j.ijbiomac.2021.07.121 34314795 10.1016/j.ijbiomac.2021.07.121

[CR31] Simões LO, Alves QL, Camargo SB et al (2021) Cardiac effect induced by Crotalus durissus cascavella venom: morphofunctional evidence and mechanism of action. Toxicol Lett 337:121–133. 10.1016/j.toxlet.2020.11.019 33238178 10.1016/j.toxlet.2020.11.019

[CR32] Stock JL, Shinjo K, Burkhardt J et al (2001) The prostaglandin E2 EP1 receptor mediates pain perception and regulates blood pressure. J Clin Invest 107:325–331. 10.1172/JCI6749 11160156 10.1172/JCI6749 PMC199184

[CR33] Sugimoto Y, Narumiya S (2007) Prostaglandin E receptors. J Biol Chem 282:11613–11617. 10.1074/jbc.R600038200 17329241 10.1074/jbc.R600038200

[CR34] Takayama K, Mitchell DH, Din ZZ et al (1994) Monomeric Re lipopolysaccharide from Escherichia coli is more active than the aggregated form in the Limulus amebocyte lysate assay and in inducing Egr-1 mRNA in murine peritoneal macrophages. J Biol Chem 269:2241–2244 8294481

[CR35] Takeuchi K, Amagase K (2018) Roles of cyclooxygenase, prostaglandin E2 and EP receptors in mucosal protection and ulcer healing in the gastrointestinal tract. Curr Pharm des 24:2002–2011. 10.2174/1381612824666180629111227 29956615 10.2174/1381612824666180629111227

[CR36] Viana MN, Leiguez E, Gutiérrez JM et al (2020) A representative metalloprotease induces PGE2 synthesis in fibroblast-like synoviocytes via the NF-$κ$B/COX-2 pathway with amplification by IL-1$β$ and the EP4 receptor. Sci Rep 10:1–15. 10.1038/s41598-020-59095-z 32094439 10.1038/s41598-020-59095-z PMC7039882

[CR37] Wang T, Fu X, Chen Q et al (2019) Arachidonic acid metabolism and kidney inflammation. Int J Mol Sci 20:1–28. 10.3390/ijms20153683 10.3390/ijms20153683 PMC6695795 31357612

[CR38] Yu L, Zhang X, Ye S et al (2022) Obesity and COVID-19: mechanistic insights from adipose tissue. J Clin Endocrinol Metab 107:1799–1811. 10.1210/clinem/dgac137 35262698 10.1210/clinem/dgac137 PMC8992328

